# The Cholesterol Metabolite Cholest-5-en-3-One Alleviates Hyperglycemia and Hyperinsulinemia in Obese (*db*/*db*) Mice

**DOI:** 10.3390/metabo12010026

**Published:** 2021-12-29

**Authors:** Koji Nagao, Nao Inoue, Kunio Suzuki, Takeshi Shimizu, Teruyoshi Yanagita

**Affiliations:** 1 Department of Biological Resource Science, Saga University, 1 Honjo-machi, Saga 840-8502, Japan; d5589@cc.saga-u.ac.jp (N.I.); yanagitt@cc.saga-u.ac.jp (T.Y.); 2 Course of Biological Science and Technology, The United Graduate School of Agricultural Sciences, Kagoshima University, Kagoshima 890-0065, Japan; 3 RIKEN Nishina Center, Wako 351-0198, Japan; baron92@nifty.com; 4 RIKEN Center for Sustainable Resource Science, Wako 351-0198, Japan; tshimizu@riken.jp

**Keywords:** 5-cholestenone, hyperglycemia, hyperinsulinemia, inflammation, obese *db*/*db* mouse

## Abstract

Dietary sterols are catabolized into various substances in the intestinal tract. Dietary 3-oxo derivatives of cholesterol and plant sterols (e.g., cholest-4-en-3-one and campest-5-en-3-one) have been shown to have anti-obesity effects. In this study, we tested whether feeding cholest-5-en-3-one (5-cholestenone), a cholesterol metabolite, to *db*/*db* mice protects them from obesity-associated metabolic disorders. In *db*/*db* mice, dietary 5-cholestenone significantly alleviated hepatomegaly and elevated serum triglyceride levels; however, the effect was not sufficient to improve hepatic steatosis and obesity. On the other hand, hyperglycemia and severe hyperinsulinemia in control *db*/*db* mice were markedly attenuated in 5-cholestenone-fed *db*/*db* mice. The production of inflammatory cytokines, such as monocyte chemoattractant protein-1, interleukin-6, and tumor necrosis factor-alpha (TNFα), was decreased, suggesting that the suppressive actions of 5-cholestenone were attributable to the alleviation of chronic inflammation in *db*/*db* mice. Additionally, 5-cholestenone showed an inhibitory effect on TNFα-induced nuclear factor kappa B (NFκB) activation in the NFκB luciferase gene reporter assay. These results suggest that obesity-induced abnormal glucose metabolism could be alleviated in 5-cholestenone-fed *db*/*db* mice by reducing the production of inflammatory cytokines through suppression of the NFκB signaling pathway.

## 1. Introduction

Diabetes mellitus is a health problem that is becoming more serious in both developing and developed countries. It is an important problem not only in the medical field but also in the socio-economic sphere. According to the data reported by the International Diabetes Federation (IDF), the global prevalence of diabetes among adults is expected to increase from 537 million in 2021 to 783 million in 2045 [1]. The increased risk of heart disease as a complication of diabetes is well known. Moreover, the incidence of atherosclerotic disease is several times higher in both type 1 and type 2 diabetes [2,3]. Type 2 diabetes mellitus is characterized by insulin resistance due to obesity. It has been shown that insulin resistance and compensatory hyperinsulinemia can easily lead to metabolic syndrome, a syndrome in which multiple atherosclerosis risks accumulate in an individual [4]. *db*/*db* mice have a missense mutation in the leptin receptor gene that causes them to overeat and develop multiple metabolic and hormonal disorders, including type 2 diabetes, which shares many features with the human metabolic syndrome [5,6].

Sterols are a general term for derivatives of the steroid skeleton that contains a hydroxy group. Cholesterol, an animal sterol, plays a role in physiological functions, but excessive accumulation thereof in the body is known to be life-threatening. Phytosterols are found in the cell membranes of plants and have a structure similar to that of cholesterol. It has been reported that phytosterols inhibit the absorption of cholesterol [7]. Moreover, it has become evident that structural isomers of steroids are generated by various types of oxidation and in vivo metabolism reactions and that the physiological effects of steroid isomers differ depending on the carbon position at which they are modified. Among them, 3β-hydroxy-5α-cholestan-6-one (6-ketocholestanol) and 3β-hydroxy-5α-cholest-8(14)-en-15-one (15-ketosterol), which have stenone structures, have been reported to have physiological effects that inhibit the synthesis of fatty acids and cholesterol, respectively [8,9]. Moreover, previous studies that chemically synthesized various 3-oxo derivatives of sterols and examined their effects on fat accumulation in rodents found that several 3-oxo derivatives of cholesterol and phytosterols (e.g., cholest-4-en-3-one (4-cholestenone) and campest-5-en-3-one (5-campestenone)) exhibited anti-obesity and lipid-lowering effects [10,11,12,13,14].

In this study, we tested whether feeding *db*/*db* mice cholest-5-en-3-one (5-cholestenone), one of the cholesterol metabolites with a stenone structure, protects them from obesity-associated metabolic disorders.

## 2. Results

### 2.1. Effects of Experimental Diets on Growth Parameters, Hepatic Triglyceride, and Serum Triglyceride

To investigate the possible effects of 5-cholestenone on the dietary parameters of mice, three test groups were set up. The *db*/*db* mice were assigned to two groups (*n* = 5) that were fed one of two diets (Table 1): a semisynthetic AIN-76 diet (CON: Control group) or a semisynthetic AIN-76 diet supplemented with 0.25% 5-cholestenone at the expense of sucrose (STN: Stenone group). In addition, C57BL/6J mice (*n* = 5), the progenitors of *db*/*db* mice, were fed the same diet as the CON group and were referred to as the NOR (Normal) group.

**Figure 1:**
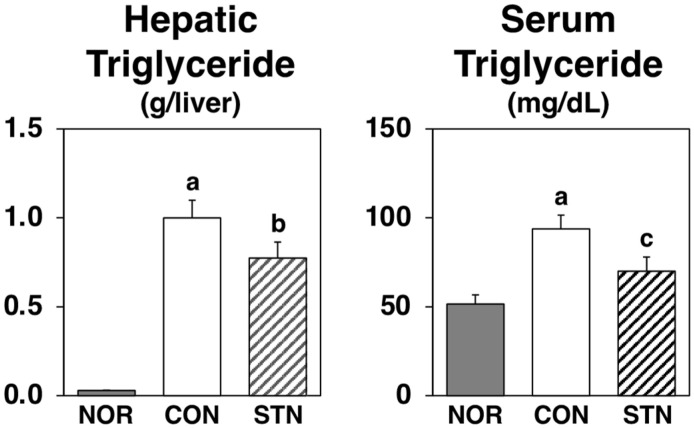
Triglyceride levels in the liver and serum of C57BL/6J and *db*/*db* mice fed experimental diets for 4 weeks. Values are expressed as the mean ± standard error (*n* = 5). ^a^ Significant difference at *p* < 0.05 between NOR and CON; ^b^ significant differences at *p* < 0.05 between NOR and STN; ^c^ significant differences at *p* < 0.05 between CON and STN.

After 4 weeks of feeding experimental diets, weight gain, food intake, liver weight, and abdominal white adipose tissue (WAT) weights were significantly higher in *db*/*db* mice fed the basal diet (CON group) when compared to the growth parameters of C57BL/6J mice fed the basal diet (NOR group), as shown in Table 2. In addition to growth parameters, liver and serum triglyceride levels were elevated, indicating that *db*/*db* mice developed fatty livers, hyperlipidemia, and obesity (Table 2, Figure 1). Although the two groups of *db*/*db* mice did not differ in initial body weight, final body weight, weight gain, or food intake, the mice fed the STN diet (STN group) had significantly reduced liver weight compared to *db*/*db* mice fed the basal diet (Table 2). Consistent with the alleviation of hepatomegaly, the STN diet showed a decreasing trend (by 23%) in triglyceride accumulation in the liver and a significant decrease (by 25%) in serum triglyceride levels (Figure 1). However, the STN diet did not alter the abdominal WAT weight of *db*/*db* mice (Table 2).

### 2.2. Effects of Experimental Diets on Glucose and Insulin Levels in the Serum

**Figure 2:**
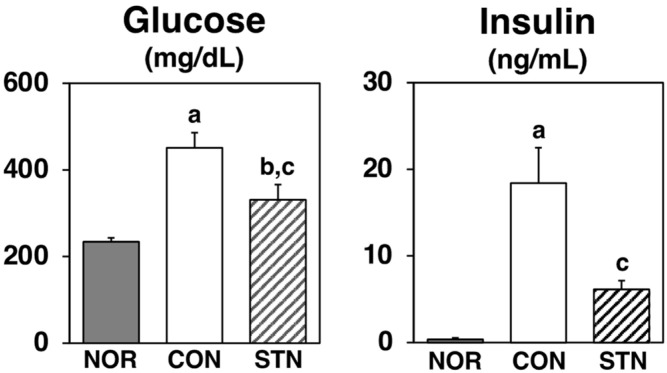
Glucose and insulin levels in the serum of C57BL/6J and *db*/*db* mice fed experimental diets for 4 weeks. Values are expressed as the mean ± standard error (*n* = 5). ^a^ Significant difference at *p* < 0.05 between NOR and CON; ^b^ significant differences at *p* < 0.05 between NOR and STN; ^c^ significant differences at *p* < 0.05 between CON and STN.

After a 4-week feeding period, the CON group had hyperglycemia and severe hyperinsulinemia. As shown in Figure 2, glucose and insulin levels in the serum were significantly decreased (by 27% and 67%, respectively) in the STN group compared to those in the CON group.

### 2.3. Effects of Experimental Diets on Adipocytokine Levels in the Serum

**Figure 3:**
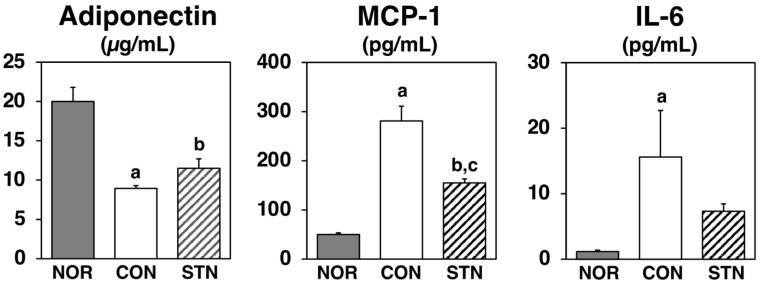
Adipocytokines levels in the serum of C57BL/6J and *db*/*db* mice fed experimental diets for 4 weeks. Values are expressed as the mean ± standard error (*n* = 5). ^a^ Significant difference at *p* < 0.05 between NOR and CON; ^b^ significant differences at *p* < 0.05 between NOR and STN; ^c^ significant differences at *p* < 0.05 between CON and STN.

Serum levels of adiponectin, which enhances insulin sensitivity, were significantly lower in the CON group compared to the NOR group; they tended to be 29% higher in the STN group compared to the CON group (Figure 3). Conversely, serum levels of monocyte chemoattractant protein-1 (MCP-1), which triggers inflammatory responses, were markedly increased in the CON group when compared to the NOR group and markedly reduced in the STN group when compared to those in the CON group (Figure 3). Moreover, serum levels of interleukin-6 (IL-6), another inflammatory adipocytokine, were markedly increased in the CON group when compared to the NOR, and they tended to be 50% lower in the STN group when compared to those in the CON group (Figure 3).

### 2.4. Effects of Experimental Diets on mRNA Levels in Perirenal White Adipose Tissue

**Figure 4:**
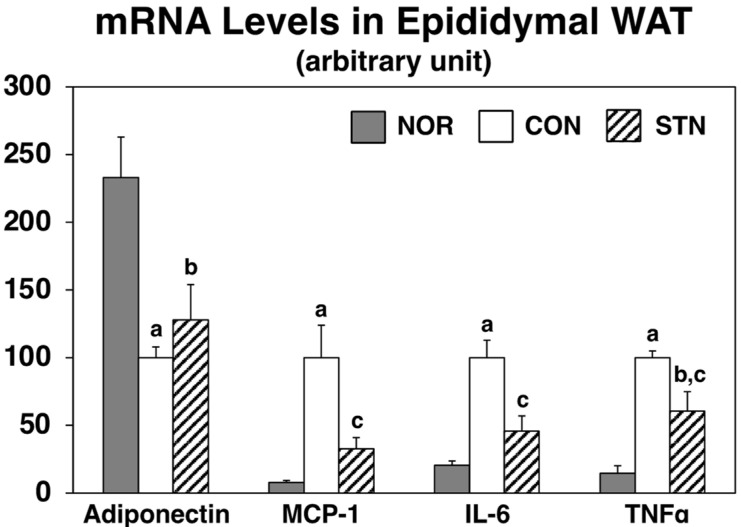
Expression levels of adipocytokine mRNAs in epididymal white adipose tissues of C57BL/6J and *db*/*db* mice fed experimental diets for 4 weeks. Values are expressed as the mean ± standard error (*n* =5). ^a^ Significant difference at *p* < 0.05 between NOR and CON; ^b^ significant differences at *p* < 0.05 between NOR and STN; ^c^ significant differences at *p* < 0.05 between CON and STN.

To examine the effects of the STN diet on adipocytokine production, we analyzed the expression of adipocytokine genes in epididymal WAT using real-time PCR (Figure 4). Consistent with serum levels, mRNA levels of adiponectin were decreased, and MCP-1 and IL-6 were increased in the CON group when compared to the NOR group. Moreover, mRNA levels of TNFα, which also triggers inflammatory responses, were markedly increased in the CON group when compared to the NOR group. In the STN group, the STN diet tended to increase adiponectin mRNA levels by 28% and significantly decreased mRNA levels of MCP-1, IL-6, and tumor necrosis factor α (TNFα) by 67%, 54%, and 39%, respectively.

### 2.5. NFκB Reporter Gene Assay In Vitro

**Figure 5:**
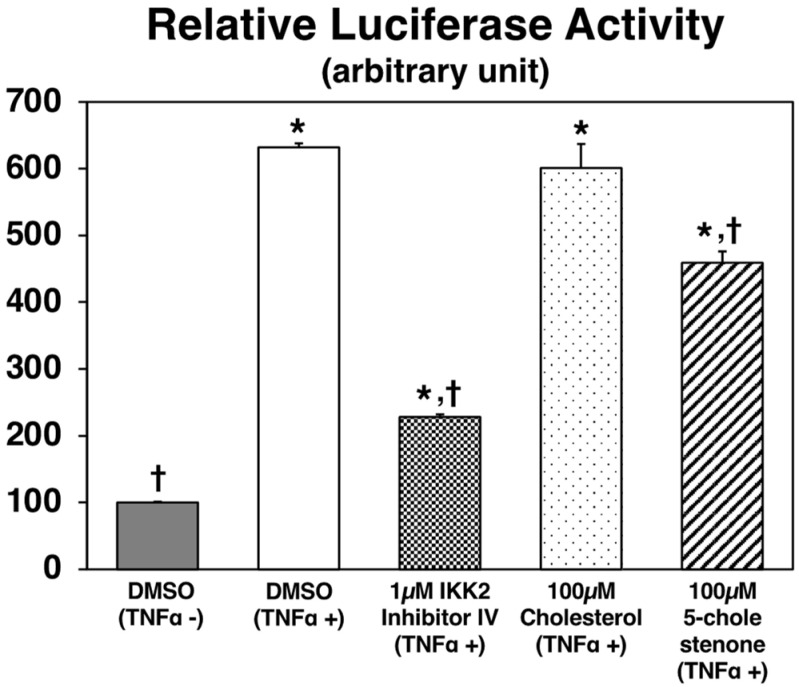
Luciferase activities in NFκB-Luc/CHO-K1 cells. Cells were treated with or without murine TNFα (10 ng/mL in DMSO), cholesterol (100 µM in DMSO), 5-cholestenone (100 µM choles-5-en-3-one in DMSO), or 1 µM IKK2 inhibitor IV. After 7 h of incubation, NFκB luciferase was detected using the ONE-Step^TM^ Luciferase assay system. Values are expressed as the mean ± standard error (*n* = 3 wells). * Significant difference at *p* < 0.05 compared to DMSO (TNFα-) treatment. † Significant difference at *p* < 0.05 compared to DMSO (TNFα+) treatment.

To further examine the bioactivity of 5-cholestenone, an nuclear factor κB (NFκB) luciferase reporter assay was carried out (Figure 5). TNFα-induced NFκB activation was not affected by a 100 µM cholesterol treatment but was significantly inhibited by a 100 µM 5-cholestenone treatment in NFκB-Luc/Chinese hamster ovary-K1 (CHO-K1) cells. Because the phosphorylation of inhibitory kappa B kinase (IKK)β induces the activation of NFκB, the inhibitory effect of 1 μM IKK2 inhibitor IV was measured as a reference. No cytotoxicity in any of the treatments was confirmed using the cell viability assay (data not shown).

## 3. Discussion

We evaluated the effect of the 5-cholestenone diet on metabolic disorders in obese *db*/*db* mice. We found that hyperglycemia and hyperinsulinemia are alleviated through the reduction of inflammatory factor production in 5-cholestenone-fed *db*/*db* mice. These effects may be attributable to the inhibitory effect of 5-cholestenone on the NFκB signaling pathway.

Obesity is known to cause a variety of metabolic disorders and has become a massive medical and social problem in modern developed countries [4,15]. Type 2 diabetes, which accounts for the majority of diabetes cases in recent years, is further characterized by insulin resistance associated with obesity [1,2,3,4,15]. There have been many studies on the anti-obesity effects of dietary components [15,16]. Previous studies have shown that a 0.5% 4-cholestenone or 0.5% 5-campestenone supplemented diet markedly reduces body fat accumulation in CDF1 mice and Sprague-Dawley rats [10,12]. In the present study, four weeks of feeding a 0.25% supplemented 5-cholestenone diet had no effect on the accumulation of abdominal fat in *db*/*db* mice (Table 2). However, considering the fact that serum triglyceride concentrations were significantly reduced (Figure 1), the anti-obesity effect of 5-cholestenone needs to be examined further in different animal models, at different doses, and for different feeding periods.

Insulin resistance and compensatory hyperinsulinemia, the major pathogenesis of type 2 diabetes, have been reported to play a role in the development of metabolic syndrome [1,2,3,4]. At present, various drugs are used for the treatment of type 2 diabetes [17]. However, the use of functional dietary ingredients to prevent and improve type 2 diabetes is desirable due to their cost-effectiveness and minimal side effects. A previous study showed that 5-campestenone acts as an antidiabetic in obese *db*/*db* mice and Zucker diabetic fatty rats [18,19]. In the present study, hyperglycemia and severe hyperinsulinemia in control *db*/*db* mice were markedly attenuated in 5-cholestenone-fed *db*/*db* mice without any undesirable effects on food intake or growth (Table 2, Figure 2). Therefore, 5-cholestenone may be a dietary additive with antidiabetic activity. 

Adipose tissue stores excess energy as fat and also secretes bioactive substances called adipocytokines [20]. Adiponectin is an abundant protein secreted from adipose tissues in rodents and humans. Many reports have shown that adiponectin leads to enhanced insulin action in vitro and in vivo, strongly suggesting its protective role against insulin resistance [20]. Glitazones are antidiabetic drugs that lead to increased expression and concurrent adiponectin levels in the blood via ligand activity to the transcription factor peroxisome proliferator-activated receptor (PPAR)-gamma [21]. In the present study, serum adiponectin levels were significantly lower in control-fed *db*/*db* mice than in C57BL/6J mice, and they were slightly higher (29%, not significant) in 5-cholestenone-fed *db*/*db* mice than in control-fed *db*/*db* mice (Figure 3). Consistent with serum levels, *adiponectin* mRNA expression levels in epididymal WAT were significantly lower in control-fed *db*/*db* mice than in C57BL/6J mice and slightly increased (28%, not significant) in 5-cholestenone-fed *db*/*db* mice than in control-fed *db*/*db* mice (Figure 4). These results suggest that the activity of 5-cholestenone as an adiponectin inducer is not as pronounced. 

Recently, it has been suggested that chronic inflammation is involved in the development of insulin resistance in metabolic syndrome [22]. Chronic inflammation is a slow and prolonged smoldering of inflammation in internal organs. The persistence of this smoldering mild inflammatory response can lead not only to altered and impaired tissue function but also to irreversible organ dysfunction in the long term due to tissue re-modeling (e.g., fibrosis). MCP-1 is a member of the CC chemokine family; it induces an inflammatory response through the recruitment of inflammatory cells [20]. Previous studies have shown that transgenic mice overexpressing *MCP-1* exhibit insulin resistance, whereas MCP-1^−/−^ knockout mice, or mice with acute downregulation of MCP-1 by expression of a dominant-negative mutant, show an improvement in insulin resistance [23]. IL-6 is also a cytokine that plays an important role in the regulation of immune responses and inflammatory reactions. Although its role is very important in maintaining homeostasis in the body, it is known to cause various pathological conditions when overproduced [20]. In the present study, serum MCP-1 and IL-6 levels were markedly higher in control *db*/*db* mice than in C57BL/6J mice. In *db*/*db* mice, MCP-1 was markedly reduced, and IL-6 was reduced by 50% (not significant) in the 5-cholestenone group compared to the control group (Figure 3). The mRNA expression of inflammatory factors (*MCP-1, IL-6, TNFα*) in epididymal WAT of *db*/*db* mice was markedly upregulated compared to that in the C57BL/6J mice (Figure 4). TNFα has been identified as a proinflammatory cytokine that kills tumor cells and microorganisms, but it has also been suggested to lower insulin sensitivity through induction of inflammatory molecules such as MCP1 and IL-6 and through the reduction of *adiponectin* expression [20,24]. In the present study, the 5-cholestenone supplemented diet significantly decreased the expression of MCP-1, IL-6, and TNFα in epididymal WAT of *db*/*db* mice. These results suggest that chronic inflammation in *db*/*db* mice causes insulin resistance and that 5-cholestenone improves hyperglycemia and hyperinsulinemia by ameliorating chronic inflammation. 

Two metabolic pathways have been proposed to explain the cholesterol catabolism by intestinal microorganisms. In one of them, cholesterol is catabolized to 5-cholestenone, then 4-cholestenone, followed by the formation of 5β-cholestan-3-one, and finally, 5β-cholestan-3β-ol (coprostanol) [25]. Hence, it is entirely likely that 5-cholestenone is easily catabolized in the intestinal tract. However, it is unknown whether 5-cholestenone catabolites (such as 4-cholestenone) are transported to the liver and adipose tissues. Moreover, the mechanisms of the anti-obesity and lipid-lowering effects of 4-cholestenone remain unknown [10,11]. Regarding the anti-obesity and lipid-lowering effects of 5-campestenone, it has been reported that it enhances hepatic β-oxidation related enzyme activity and mRNA expression through its ligand activity for PPARα, and consequently, it increases ketone body production, thereby inhibiting fatty acid biosynthesis [12,13]. NFκB is a transcription factor that regulates the expression of many genes that cause inflammation, including MCP-1, IL-6, and TNFα [26]. Administration of inhibitors of the NFκB activating enzyme (IKK2: inhibitory kappa-B kinase subunit beta) suppresses insulin resistance in diabetic mice [27], suggesting that suppression of NFκB signaling is a promising therapeutic target for the prevention and alleviation of inflammatory diseases, including metabolic syndrome. Additionally, chronic inflammation in metabolic syndrome has been shown to cause functional abnormalities not only in adipose tissue but also in the intestinal tract, and these tissues partake in metabolic crosstalk (gut-adipose tissue axis) [28]. Dietary factors with anti-inflammatory activity have been reported to improve chronic inflammation throughout the body by regulating the gut microbiota [29]. In the present study, in vitro reporter gene assays showed that 5-cholestenone significantly inhibited NFκB activation. Thus, the anti-inflammatory properties of 5-cholestenone via suppression of the NFκB signaling pathway in the intestinal tract may be at least partly responsible for the alleviation of chronic inflammation in *db*/*db* mice.

**Figure 6:**
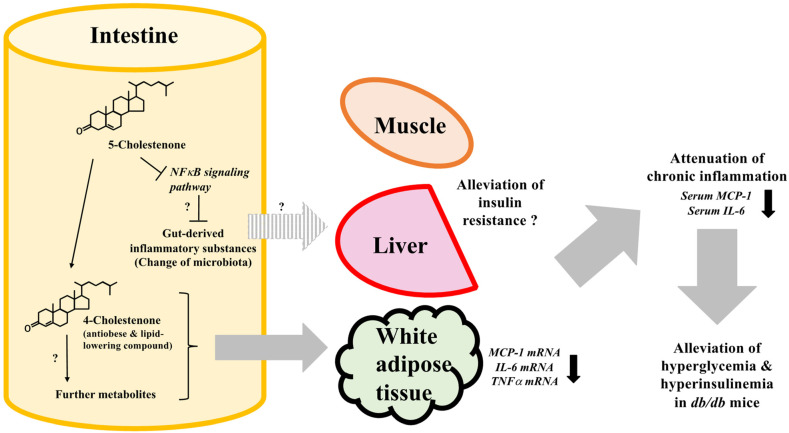
The proposed molecular mechanism by which 5-cholestenone affects metabolic disorders in *db*/*db* mice.

One of the limitations of our study is that various molecules and pathways other than those investigated in this study are involved in obesity-induced metabolic aberrations, including insulin resistance. For example, phosphotidylinsositol-3-kinase signaling pathway is a crucial modulator of glucose metabolism. This signal transduction pathway is stimulated by leptin, an appetite-regulating adipocytokine [30]. Moreover, visfatin, another adipocytokine that acts as a pre-B cell colony-enhancing factor, is known to upregulate other inflammatory cytokines [31]. Additionally, integral membrane protein GLUTs, the principal glucose transporters involved in glucose uptake [32], and the caspase cascade are also involved in apoptosis and inflammation [33,34,35]. Studies have established their correlation with the induction of insulin resistance [30,31,32,33,34,35]. In order to demonstrate the mechanism of the effects of 5-cholestenone, further research is needed, including (1) analysis of the kinetics of 5-cholestenone and its metabolites in the body, (2) analysis of changes in the gut microbiota and gut-derived inflammatory substances, (3) comprehensive analysis of the 5-cholestenone-induced variations in adipocytokine profiles, and (4) detailed examination of the mechanism by which 5-cholestenone influences insulin sensitivity (Figure 6). By investigating these aspects, the intake of 3-oxo derivatives of sterols and/or anti-inflammatory compounds can be established as an effective therapeutic strategy in the prevention and treatment of obesity-associated metabolic disorders.

In conclusion, the present study shows that 5-cholestenone may have an NFκB inhibitory effect, suggesting that a diet supplemented with 5-cholestenone may potentially alleviate obesity-induced metabolic disorders through its action as an anti-inflammatory agent in the metabolic syndrome.

## 4. Materials and Methods

### 4.1. Animals

All aspects of the experiment were conducted in accordance with the guidelines provided by the ethical committee for experimental animal care at Saga University (certificate number: 22-042-1). Five-week-old male C57BL/6J and *db*/*db* mice were purchased from Japan SLC (Shizuoka, Japan). The mice were housed individually in plastic cages in a temperature-controlled room (24 °C) under a 12 h light/dark cycle. The basal semisynthetic diets were prepared according to the recommendations of the American Institute of Nutrition (AIN-76) [36] (Table 1). Cholest-5-en-3one (5-cholestenone) was chemically synthesized from cholesterol according to the method of Parish et al. [37] and purified by recrystallization. The *db*/*db* mice were assigned to two groups (*n* = 5) that were fed one of two diets (Table 1): a semisynthetic AIN-76 diet (CON group) or a semisynthetic AIN-76 diet supplemented with 0.25% 5-cholestenone at the expense of sucrose (STN group). C57BL/6J mice (*n* = 5), the progenitors of *db*/*db* mice, were fed the same diet as the *db*/*db* mice in the control group (NOR group). The mice received the diets ad libitum using Rodent CAFE (KBT Oriental, Saga, Japan) for 4 weeks. At the end of the feeding period, the mice were sacrificed by exsanguination from the heart under isoflurane anesthesia after a 9 h starvation period. White adipose tissue (WAT) and livers were excised immediately, and the serum was separated from the blood. 

### 4.2. Measurement of Hepatic Triglyceride Levels

Total liver lipids were extracted using the method described by Folch et al. [38]. Liver triglyceride concentrations were quantified using the method of Fletcher et al. [39]. 

### 4.3. Serum Parameters

Serum triglyceride and glucose levels were measured using commercial enzyme assay kits (Wako Pure Chemicals, Tokyo, Japan). Serum insulin (LBIS rat insulin ELISA kit, Shibayagi, Gunma, Japan), adiponectin (mouse/rat adiponectin ELISA kit, Otsuka Pharmaceutical, Tokyo, Japan), MCP-1 (Mouse CCL2/JE/MCP-1 Quantikine ELISA kit, R&D Systems, Minneapolis, MN, USA) and IL-6 (Mouse IL-6 Quantikine ELISA kit, R&D Systems, Minneapolis, MN, USA) levels were measured using the indicated commercial mouse ELISA kits.

### 4.4. Analysis of mRNA Expression in Perirenal WAT

Total RNA was extracted from 100 mg of epididymal WAT using the RNeasy Lipid Tissue Mini Kit (Qiagen, Tokyo, Japan). TaqMan Universal PCR Master Mix (Applied Biosystems, Tokyo, Japan) and Assay-on-Demand, Gene Expression Products (Mn00456425_m1 for adiponectin, Mn00446190_m1 for *IL-6*, Mn00443258_m1 for *TNFα*, Mn00441242_m1 for *MCP1*, and Mm04277571_s1 for *18S RNA*, all from Applied Biosystems, Tokyo, Japan) were used for quantitative RT-PCR analysis of adiponectin, MCP1, TNFα, and 18S RNA expression in epididymal WAT. Amplifications were carried out using an ABI Prism 7000 RT-PCR sequence detection system (Applied Biosystems, Tokyo, Japan).

### 4.5. In Vitro NFκB Reporter Gene Assay

In the NFκB-Luc/CHO-K1 cell line (BPS Bioscience, San Diego, CA, USA), fLUC expression is controlled by the NFκB response element located upstream of the TATA promoter and is suitable for monitoring the activity of the NFκB transcription factor through luminescence readout. Cells were seeded at 1.0 × 10^5^ cells/well in a 96-well plate overnight in a complete growth medium (F-12K with 10% FBS and G418). Cells were treated with or without murine TNFα (10 ng/mL, BPS Bioscience, San Diego, CA, USA) in a growth medium and incubated for 7 h at 37 °C before the addition of luciferin, according to the manufacturer’s protocol (ONE-Step^TM^ Luciferase assay system, BPS Bioscience, San Diego, CA, USA). TNFα-stimulated cells were also treated with dimethyl sulfoxide (DMSO, 1% *v*/*v*), cholesterol (100 µM in DMSO), 5-cholestenone (100 µM cholest-5-en3-one in DMSO), or IKK2 inhibitor IV (1 µM, EMD Biosciences Inc., La Jolla, CA, USA). Luminescence was read using a luminometer (Lumat LB9507, Berthold Japan K.K., Tokyo, Japan), and readings were normalized to wells that contained only the medium to obtain the relative luminescence units. To evaluate the cytotoxicity of each treatment, water-soluble disulfonated tetrazolium salt synthetic activity [40] was determined using a Cell Counting Kit-8 (Dojindo Laboratories, Kumamoto, Japan).

### 4.6. Statistical Analysis

All data values are expressed as the mean ± standard error. To assess differences among the three groups, data were analyzed by one-way ANOVA, and all differences were analyzed by the Fisher’s LSD post hoc test using the KaleidaGraph software version 4.5 (Synergy Software, Reading, PA, USA). Differences were considered significant at *p* < 0.05.

## Figures and Tables

**Table 1:** Composition of experimental diets.

Ingredients	Basal Diet	STN Diet ^b^
	%	
Casein	20	20
Cornstarch	15	15
Cellulose	5	5
Mineral mixture ^a^	3.5	3.5
Vitamine mixture ^a^	1	1
DL-Methionine	0.3	0.3
Choline bitartrate	0.2	0.2
Corn oil	7	7
Cholest-5-en-3-one (5-cholestenone)	-	0.25
Sucrose	48	47.75

^a^ AIN-76, ^b^ STN (5-cholestenone).

**Table 2:** Effect of experimental diet on growth parameters in C57BL/6J and *db*/*db* mice.

	NOR	CON	STN
Initial body weight (g)	22.2 ± 0.2	26.8 ± 0.2 ^a^	27.1 ± 0.2 ^b^
Final body weight (g)	23.4 ± 0.3	37.0 ± 0.5 ^a^	38.1 ± 0.4 ^b^
Body weight gain (g)	1.24 ± 0.21	10.1 ± 0.4 ^a^	11.0 ± 0.4 ^b^
Food intake (g)	74.7 ± 0.6	103 ± 1 ^a^	103 ± 2 ^b^
Liver weight (g/100 g body weight)	3.90 ± 0.03	7.45 ± 0.48	5.98 ± 0.56 ^b,c^
White adipose tissue weight (g/100 g body weight)			
Total	2.63 ± 0.17	7.22 ± 0.27 ^a^	6.95 ± 0.31 ^b^
Epididymal	1.85 ± 0.09	4.49 ± 0.12 ^a^	4.32 ± 0.13 ^b^
Perirenal	0.781 ± 0.082	2.73 ± 0.18 ^a^	2.63 ± 0.27 ^b^

^a^ Significant difference at *p* < 0.05 between NOR and CON; ^b^ significant differences at *p* < 0.05 between NOR and STN; ^c^ significant differences at *p* < 0.05 between CON and STN.

## Data Availability

The datasets used and/or analyzed during the current study are available from the corresponding author, because of it’s usage in the ongoing study.

## References

[1] International Diabetes Federation (2021). IDF Diabetes Atlas.

[2] Fisher M. (2004). Diabetes and atherogenesis. Heart.

[3] Varughese G.I., Tomson J., Lip G.Y. (2005). Type 2 diabetes mellitus: A cardiovascular perspective. Int. J. Clin. Pract..

[4] Eckel R.H., Grundy A.M., Zimmet P.Z. (2005). The metabolic syndrome. Lancet.

[5] Chen H., Charlat O., Tartaglia L.A., Woolf E.A., Weng X., Ellis S.J., Lakey N.D., Culpepper J., More K.J., Breitbart R.E. (1996). Evidence that the diabetes gene encodes the leptin receptor: Identification of a mutation in the leptin receptor gene in *db*/*db* mice. Cell.

[6] Lee G.H., Proenca R., Montez J.M., Carroll K.M., Darvishzadah J.G., Lee G.I., Friedman J.M. (1996). Abnormal splicing of the leptin receptor in diabetic mice. Nature.

[7] Makhmudova U., Schulze P.C., Lütjohann D., Weingärtner O. (2021). Phytosterols and Cardiovascular Disease. Curr. Atheroscler. Rep..

[8] Shirouchi B., Yanagi S., Okawa C., Koga M., Sato M. (2020). 6-Ketocholestanol suppresses lipid accumulation by decreasing FASN gene expression through SREBP-dependent regulation in HepG2 cells. Cytotechnology.

[9] Schmidt R.J., Ficorilli J.V., Zhang Y., Bramlett K.S., Beyer T.P., Borchert K., Dowless M.S., Houck K.A., Burris T.P., Eacho P.I. (2006). A 15-ketosterol is a liver X receptor ligand that suppresses sterol-responsive element binding protein-2 activity. J. Lipid Res..

[10] Suzuki K. (1993). Anti-obesity effect of cholest-4-en-3-one, an intestinal catabolite of cholesterol, on mice. J. Nutr. Sci. Vitaminol..

[11] Suzuki K., Shimizu T., Nakata T. (1998). The cholesterol metabolite cholest-4-en-3-one and its 3-oxo derivatives suppress body weight gain, body fat accumulation and serum lipid concentration in mice. Bioorg. Med. Chem. Lett..

[12] Ikeda I., Konno R., Shimizu T., Ide T., Takahashi N., Kawada T., Nagao K., Inoue N., Yanagita T., Hamada T. (2006). Campest-5-en-3-one, an oxidized derivative of campesterol, activates PPARalpha, promotes energy consumption and reduces visceral fat deposition in rats. Biochim. Biophys. Acta.

[13] Tamaru S., Suzuki Y., Sakono M., Fukuda N., Ikeda I., Konno R., Shimizu T., Suzuki K. (2006). Dietary 5-campestenone (campest-5-en-3-one) enhances fatty acid oxidation in perfused rat liver. J. Nutr. Sci. Vitaminol..

[14] Suzuki K., Konno R., Shimzu T., Nagashima T., Kimura A. (2007). A fermentation product of phytosterol including campestenone reduces body fat storage and body weight gain in mice. J. Nutr. Sci. Vitaminol..

[15] Boccellino M., D’Angelo S. (2020). Anti-Obesity Effects of Polyphenol Intake: Current Status and Future Possibilities. Int. J. Mol. Sci..

[16] Jiang H., Zhang W., Li X., Xu Y., Cao J., Jiang W. (2021). The anti-obesogenic effects of dietary berry fruits: A review. Food Res. Int..

[17] Leitner D.R., Frühbeck G., Yumuk V., Schindler K., Micic D., Woodward E., Toplak H. (2017). Obesity and Type 2 Diabetes: Two Diseases with a Need for Combined Treatment Strategies—EASO Can Lead the Way. Obes. Facts.

[18] Suzuki K., Tanaka M., Konno R., Kaneko Y. (2002). Effects of 5-campestenone (24-methylcholest-5-en-3-one) on the type 2 diabetes mellitus model animal C57BL/KsJ-db/db mice. Horm. Metab. Res..

[19] Konno R., Kaneko Y., Suzuki K., Matsui Y. (2005). Effect of 5-Campestenone (24-methylcholest-5-en-3-one) on Zucker diabetic fatty rats as a type 2 diabetes mellitus model. Horm. Metab. Res..

[20] Cao H. (2014). Adipocytokines in obesity and metabolic disease. J. Endocrinol..

[21] Chang E., Park C.Y., Park S.W. (2013). Role of thiazolidinediones, insulin sensitizers, in non-alcoholic fatty liver disease. J. Diabetes Investig..

[22] Lumeng C.N., Saltiel A.R. (2011). Inflammatory links between obesity and metabolic disease. J. Clin. Investig..

[23] Kanda H., Tateya S., Tamori Y., Kotani K., Hiasa K., Kitazawa R., Kitazawa S., Miyachi H., Maeda S., Egashira K. (2006). MCP-1 contributes to macrophage infiltration into adipose tissue, insulin resistance, and hepatic steatosis in obesity. J. Clin. Investig..

[24] Wang B., Jenkins J.R., Trayhurn P. (2005). Expression and secretion of inflammation-related adipokines by human adipocytes differentiated in culture: Integrated response to TNF-alpha. Am. J. Physiol. Endocrinol. Metab..

[25] Juste C., Gérard P. (2021). Cholesterol-to-Coprostanol Conversion by the Gut Microbiota: What We Know, Suspect, and Ignore. Microorganisms.

[26] Ghosh S., Karin M. (2002). Missing pieces in the NF-κB puzzle. Cell.

[27] Kim J.K., Kim Y.J., Fillmore J.J., Chen Y., Moore I., Lee J., Yuan M., Li Z.W., Karin M., Perret P. (2001). Prevention of fat-induced insulin resistance by salicylate. J. Clin. Investig..

[28] Konrad D., Wueest S. (2014). The gut-adipose-liver axis in the metabolic syndrome. Physiology.

[29] Morais C.A., de Rosso V.V., Estadella D., Pisani L.P. (2016). Anthocyanins as inflammatory modulators and the role of the gut microbiota. J. Nutr. Biochem..

[30] Yadav A., Kataria M.A., Saini V., Yadav A. (2013). Role of leptin and adiponectin in insulin resistance. Clin. Chim. Acta.

[31] Erten M. (2021). Visfatin as a Promising Marker of Cardiometabolic Risk. Acta Cardiol. Sin..

[32] Teixeira G.P., Faria R.X. (2021). Influence of purinergic signaling on glucose transporters: A possible mechanism against insulin resistance?. Eur. J. Pharmacol..

[33] Tian C., Tuo Y.L., Lu Y., Xu C.R., Xiang M. (2020). The Role of CARD9 in Metabolic Diseases. Curr. Med. Sci..

[34] Haneklaus M., O’Neill L.A. (2015). NLRP3 at the interface of metabolism and inflammation. Immunol. Rev..

[35] Tomita T. (2016). Apoptosis in pancreatic β-islet cells in Type 2 diabetes. Bosn. J. Basic. Med. Sci..

[36] American Institute of Nutrition (1977). Report of the American Institute of Nutrition ad hoc committee on standards for nutritional studies. J. Nutr..

[37] Parish E.J., Luo C., Parish S., Heidepriem R.W. (1992). Selective oxidation of steroidal homoallylic alcohols using pyridium chlorochromate (PPC). Synth. Commun..

[38] Folch J., Lees M., Sloane-Stanley G.H. (1957). A simple method for the isolation and purification of total lipids from animal tissues. J. Biol. Chem..

[39] Fletcher M.J. (1968). A colorimetric method for estimating serum triglycerides. Clin. Chim. Acta.

[40] Ishiyama M., Miyazono Y., Sasamoto K., Ohkura Y., Ueno K. (1997). A highly water-soluble disulfonated tetrazolium salt as a chromogenic indicator for NADH as well as cell viability. Talanta.

